# Rapid diagnostic tests for the detection of recent dengue infections: An evaluation of six kits on clinical specimens

**DOI:** 10.1371/journal.pone.0249602

**Published:** 2021-04-01

**Authors:** Kok-Siang Yow, Joel Aik, Eugene Yong-Meng Tan, Lee-Ching Ng, Yee-Ling Lai

**Affiliations:** Environmental Health Institute, National Environment Agency, Singapore, Singapore; CEA, FRANCE

## Abstract

**Introduction:**

Early and rapid confirmation of dengue infections strengthens disease surveillance program and are critical to the success of vector control measures. Rapid diagnostics tests (RDTs) are increasingly used to confirm recent dengue infections due to their ease of use and short turnaround time for results. Several studies undertaken in dengue-endemic Southeast Asia have reported the performance of RDTs against enzyme-linked immunosorbent assay (ELISA), reverse transcriptase polymerase chain reaction (RT-PCR) and virus isolation methods. However, few studies have compared multiple RDTs for the detection of dengue NS1 antigen and IgM antibody in a single combo cassette. We evaluated six RDTs in Singapore for their utility in routine clinical testing to detect recent dengue infections.

**Methods:**

The evaluation comprised two phases. The first phase sought to determine each RDT’s specificity to dengue NS1 and IgM using zika and chikungunya virus supernatant and zika convalescent samples. RDTs that cross-reacted with zika or chikungunya were not further tested in phase 2. The second phase sought to determine the sensitivity and specificity of the remaining RDTs to dengue NS1 and IgM using pre-characterised dengue specimens and non-dengue/chikungunya febrile clinical specimens.

**Results:**

None of the RDTs cross-reacted with zika IgM in Phase 1. Truquick and Quickprofile cross reacted with zika and chikungunya viruses and were not evaluated thereafter. Standard Q had the highest dengue NS1 and IgM sensitivity at 87.0% and 84.3% respectively whereas Bioline (68.5%) and Multisure (58.3%) had the lowest dengue NS1 and IgM sensitivity respectively. Combining dengue NS1/IgM detection results greatly improved the RDT ability to detect recent dengue infection; Standard Q had the highest sensitivity at 99.1% while Multisure had the lowest at 92.6%. All the RDTs were highly specific for dengue NS1 and IgM (96.7% to 100%). All the RDTs had high positive predictive values (98.4% to 100%) for NS1, IgM and combined NS1/IgM parameters whereas Standard Q had the highest negative predictive values at 68.2% (NS1), 63.8% (IgM) and 96.8% (NS1/IgM). For the RDTs, detection of NS1 declined from acute to convalescent phase of illness whereas IgM detection rate gradually increased over time.

**Conclusion:**

In our study, several RDTs were evaluated for their diagnostic accuracy and capability in detecting recent dengue infection. Standard Q demonstrated a high degree of diagnostic accuracy and capability in the detection of NS1 and IgM biomarkers. RDTs can provide rapid and accurate confirmation of recent dengue infections and augment dengue surveillance and control programmes. Further studies are required to assess the usefulness of these RDTs in other epidemiology settings.

## 1. Introduction

Dengue is one of the most important infectious diseases in tropical and subtropical regions of the world causing 100 to 200 million infections per year [1]. These infections represent a significant disease burden in endemic countries and without a safe and effective dengue vaccine, early diagnosis of dengue infection is critical for effective disease control, patient management and etiological investigation [2,3].

There are four dengue serotypes (DENV-1 to 4) which cause illness in humans. The dengue virus infection can be asymptomatic or may cause undifferentiated febrile illness, dengue fever or dengue haemorrhagic fever including dengue shock syndrome. The clinical manifestation of dengue infection depends on factors such as the immune status, age and underlying medical condition of the host [4]. An accurate laboratory diagnosis of a recent dengue infection is essential as a delay in diagnosis increases the risk of severe dengue and could lead to a poor disease outcome [5].

Traditionally, dengue diagnosis can be achieved via virus isolation, immunological-based tests or the molecular-based polymerase chain reaction (PCR). However, these techniques are costly, have a long turnaround time and require specialised equipment and training [6]. In recent years, dengue rapid diagnostic tests (RDTs) have been developed commercially to detect dengue NS1 antigen, IgM and IgG antibodies to dengue virus from whole blood, serum or plasma. These RDTs are based on the immunochromatographic method and are presented in a lateral flow cassette that draws specimen via capillary action and the results can be obtained in less than 30 minutes [7].

NS1, which is a highly conserved glycoprotein among Flaviviruses such as Zika, Dengue and Japanese encephalitis, can be found in day one and up to day nine after onset of fever in both primary and secondary dengue infected patients [8,9]. IgM is detectable at day three to five of illness and can persist up to three months whereas IgG appear by day 14 and persist for life [10]. In patients with secondary dengue infection, IgG rises within two days after illness and may not be accompanied by a positive NS1 or IgM result. Therefore, simultaneous testing of NS1, IgM and IgG in a single cassette can be used for preliminary dengue diagnosis from acute to convalescent stage of dengue infection.

Manufacturers have reported the performance of dengue RDTs, but past evaluations have shown varied, including poor performance, among different dengue RDTs [11,12]. This presents a challenge for healthcare providers and authorities seeking to rely on rapid diagnostic tests for the purpose of clinical management and effective disease control. In this study, we aimed to assess the diagnostic accuracy and capability of six dengue RDTs to dengue NS1 and IgM biomarkers for the confirmation of recent dengue infections.

## 2. Materials and methods

### 2.1 Ethics statement

The Environmental Health Institute (EHI) management committee chaired by Dr Lee-Ching Ng, Group Director of EHI, waived the need for informed consent as the archived specimens were fully anonymized prior to the start of the study. The ethical approval can be found under TS233.

### 2.2 Study area

In Singapore, dengue is endemic due to the presence of the primary dengue vector *Aedes aegypti* mosquito that inhabited urban areas. The dengue epidemic cycle in Singapore in the last two decades, oscillated between DENV-2 and DENV-1 even though all four DENV serotypes are reported [13].

Under the Infectious Diseases Act (IDA), all clinical laboratories and medical practitioners are legally required to notify the Ministry of Health (MOH) of all clinical or laboratory diagnosed dengue cases. The Environmental Health Institute (EHI), a public health laboratory at the National Environment Agency (NEA) conducts dengue virus surveillance including the identification of the serotype and genotype of the dengue virus. In EHI, all suspected dengue specimens collected through the Institute’s virus surveillance program are tested for NS1 and IgM using a suitable RDT. In the World Health Organization (WHO) Dengue guidelines, a single serum specimen that are positive for IgM or IgG with a HI titre of more than 1280 are indicative of probable dengue infection while detection of NS1 confirms early dengue infection [4]. In line with the WHO guidelines, EHI notifies the MOH of all patient cases tested to be either NS1 and/or IgM positive. The RDT used in EHI is a qualitative method and is unable to differentiate acute secondary dengue infection from past dengue infection based on a single IgG result. Therefore, MOH is not notified for cases with only an IgG result.

### 2.3 Dengue RDTs

The six dengue RDTs evaluated in this study are qualitative tests that detect dengue NS1 antigen, IgM and IgG to dengue virus based on the immunochromatographic method. Results interpretation for all the RDTs were done visually by the naked eye, without the need of any specialised equipment or analyser. The characteristic of each RDT is summarized in Table 1.

**Table 1:** Characteristics of the six rapid diagnostics tests (RDTs).

Manufacturer	Kit name	Diagnostic panels	Recommended Specimen Type(s)	Specimen volume (μl)	Time required (min)
Standard Diagnostics	Bioline Dengue Duo	NS1, IgM, IgG	Whole blood, plasma & serum	110	20
Wells Bio, Inc.	careUS™ Dengue Combo NS1 & IgM/IgG	NS1, IgM, IgG	Whole blood, plasma & serum	70 (plasma/serum), 100 (whole blood)	20
SD Biosensor	Standard Q^TM^ Dengue Duo	NS1, IgM, IgG	Whole blood, plasma & serum	110	20
MP Biomedicals	MULTISURE^®^ Dengue Ab/Ag Rapid Test	NS1, IgM, IgG, IgA	Whole blood, plasma & serum	25 (plasma/serum), 20 (whole blood)	25
Meridian Bioscience, Inc.	Truquick™ DENG IgG/IgM/NS1	NS1, IgM, IgG	Whole blood, plasma & serum	80 (plasma/serum), 85 (whole blood)	20
LumiQuick Diagnostics, Inc.	QuickProfile™ Dengue NS1 Antigen & IgG/IgM Antibody Duo Panel	NS1, IgM, IgG	Whole blood, plasma & serum	155	20

All testing was performed by trained analysts who undergo annual proficiency testing and interpreted according to the manufacturer’s instructions at the Environmental Health Institute, National Environment Agency of Singapore. For ambiguous results, a second confirmation were sought from another analyst to confirm the final result in order to reduce observer bias.

### 2.4 Study design

**Fig 1:**
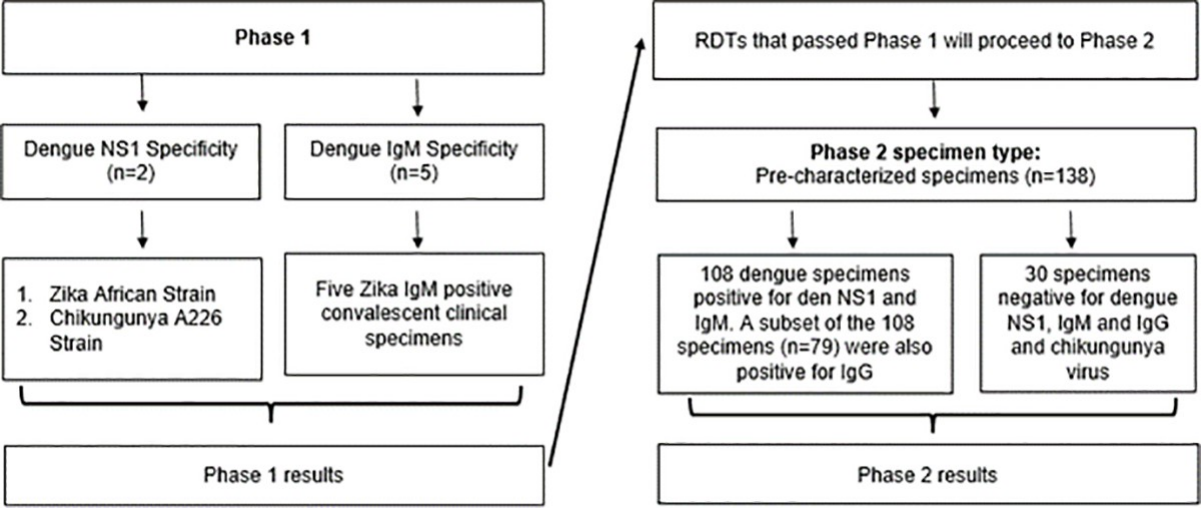
Overview of the evaluation study.

The evaluation was conducted in two phases to overcome the issue of limited number of pre-characterised specimens available for testing. The first phase acted as a screening round to eliminate RDTs that were not specific to dengue NS1 and/or IgM and therefore, only RDTs with promising results were evaluated using the pre-characterised specimens (Fig 1).

The objective of the first phase was to determine dengue NS1 and IgM specificity using zika and chikungunya virus supernatant and zika convalescent specimens. RDTs that cross reacted with either zika or chikungunya NS1 or zika IgM were eliminated in this round. Zika and Chikungunya virus was chosen for specificity testing because they co-circulate with dengue in Singapore [14,15].

In phase 2, the sensitivity and specificity of the remaining RDTs were tested using pre-characterised specimens. In this study, only the NS1 and IgM biomarkers were considered for confirmation of recent dengue infection. As described in a 2004 study, IgG have a high propensity to cross react with other related flaviviruses’ antibody and it is difficult to differentiate between present, recent or past dengue infection based on IgG alone due to its long persistence [16]. Without the ability to accurately differentiate recent dengue infection from past infection, the IgG results of the RDTs do not directly aid in early dengue diagnosis, clinical management and vector control. Therefore, we did not evaluate the RDTs’ IgG test panel with regard to their utility in confirming recent dengue infections. Nonetheless, the IgG detection results are available as supplementary data in the annex.

### 2.5 Characteristic of specimens used in the study

#### 2.5.1 Phase 1

To examine the RDTs’ specificity for dengue biomarkers, virus supernatant of the African strain of zika virus (GenBank accession number: NCBI NC_012532) and the A226 strain of Chikungunya virus (GenBank accession number: EU441882) were used to assess dengue NS1 specificity while five zika convalescent samples were used to determine dengue IgM specificity.

#### 2.5.2 Phase 2

To measure the RDTs’ sensitivity in detecting dengue biomarkers, 138 pre-characterised serum specimens collected through a study conducted from October 2011 to May 2012 were used. These sera were collected from patients with undifferentiated febrile illness with a median age of 33 years old. The serum specimens were stored in a -80°C non-frost-freeze freezer and were not freeze-thawed over the years prior to the start of the study. Previous studies have reported that antigens and antibodies were still functional when stored at -20°C in a non-frost-free freezer [17–20].

Out of the 138 pre-characterised serum specimens, a total of 108 were positive for both dengue NS1 and IgM. A subset of the 108 specimens (n = 79) were positive for IgG. The 108 pre-characterised dengue specimens consisted of 3.7% DENV-1 [4/108], 91.67% DENV-2 [99/108], 2.78% DENV-3 [3/108] and 1.85% DENV-4 [2/108]. The fever duration of the positive specimens, including the subset of IgG positive serum specimens, ranged from 4 to 11 days and had a median fever duration of 7 days. A positive dengue NS1 and/or IgM result was used to confirm dengue infection in the combined dengue NS1/IgM test panel. In addition to the virus supernatants, 30 pre-characterised non-dengue and non-chikungunya specimens were collected from patients with undifferentiated febrile illness for specificity testing. The fever duration of the negative specimens ranged from 3 to 9 days and had a median fever duration of 5 days. The characteristics of the specimens used in the study is summarized in Table 2.

**Table 2:** Characteristics of the specimens used in this study.

Serum Specimens	Total number of specimens	Days of illness					Reference tests used:
		≤5 days	6 days	7 days	8 days	≥9 days	
Dengue 1	4	1	0	1	1	1	RT-PCR and IgM/IgG ELISA
Dengue 2	99	20	18	26	17	18	
Dengue 3	3	0	1	1	1	0	
Dengue 4	2	0	1	1	0	0	
**Subtotal**	**108**	**21**	**20**	**29**	**19**	**19**	
Non dengue	30	16	6	4	3	1	RT-PCR and IgM/IgG ELISA
**Total**	**138**	**37**	**26**	**33**	**22**	**20**	

The reference tests in the study included a two-stage RT-PCR comprising of an initial screening using SYBR green followed by the tetraplex probe-based serotype specific assay. The serological assays used were: Platelia^™^ NS1 ELISA, Panbio^®^ Dengue IgG Indirect, IgG Capture and IgM Capture ELISAs. Viral isolation in C6/36 cells and confirmatory immunofluorescence testing for serotype identification were performed as described [4,21].

The specimens were classified as a confirmed case for recent dengue infection when tested to be positive by PCR or NS1, or with IgM or IgG Indirect seroconversion, or with a four-fold rise in IgG Indirect in the convalescent specimen or were positive by viral isolation. Specimens that were negative by PCR, NS1, IgM but maybe IgG Indirect positive without four-fold rise in titre in the convalescent specimen were classified as negative for recent dengue infection. Chikungunya RT-PCR were performed on all the pre-characterised samples to screen for acute chikungunya infection [21].

### 2.6 Data analysis

Sensitivity and specificity indices with a 95% confidence interval (CIs) were performed using the proportional test in R software. Positive predictive value (PPV) and negative predictive values (NPV) indices with a 95% confidence interval (CIs) were calculated as described [22].

## 3. Results

### 3.1 Phase 1 –Dengue NS1 and IgM specificity

The phase 1 evaluation results are summarized in Tables 3 and 4. Truquick and QuickProfile cross reacted with the African zika virus supernatant at 10^3 pfu/ml and with the A226 strain of the chikungunya virus supernatant at 10^2 pfu/ml. They were thus excluded from the next study phase. None of the RDTs cross reacted with zika convalescent specimens.

**Table 3:** Phase 1 evaluation results using zika IgM positive convalescent samples.

Test	Specificity for dengue IgM antibody using zika IgM positive convalescent samples				
	Sample 1	Sample 2	Sample 3	Sample 4	Sample 5
**Bioline**	Negative	Negative	Negative	Negative	Negative
**careUS**	Negative	Negative	Negative	Negative	Negative
**Standard Q**	Negative	Negative	Negative	Negative	Negative
**Multisure**	Negative	Negative	Negative	Negative	Negative
**Truquick**	Negative	Negative	Negative	Negative	Negative
**QuickProfile**	Negative	Negative	Negative	Negative	Negative

**Table 4:** Phase 1 evaluation results using virus supernatant.

Test	Specificity for dengue NS1 antigen using virus supernatant	
	Zika African virus, 10^3 pfu/mL (GenBank: NCBI NC_012532)	Chikungunya A226 virus, 10^2 pfu/ml (GenBank: EU441882)
**Bioline**	Negative	Negative
**careUS**	Negative	Negative
**Standard Q**	Negative	Negative
**Multisure**	Negative	Negative
**Truquick**	Positive	Positive
**QuickProfile**	Positive	Positive

### 3.2 Phase 2 –Diagnostic accuracy and capability of the RDTs

**Fig 2:**
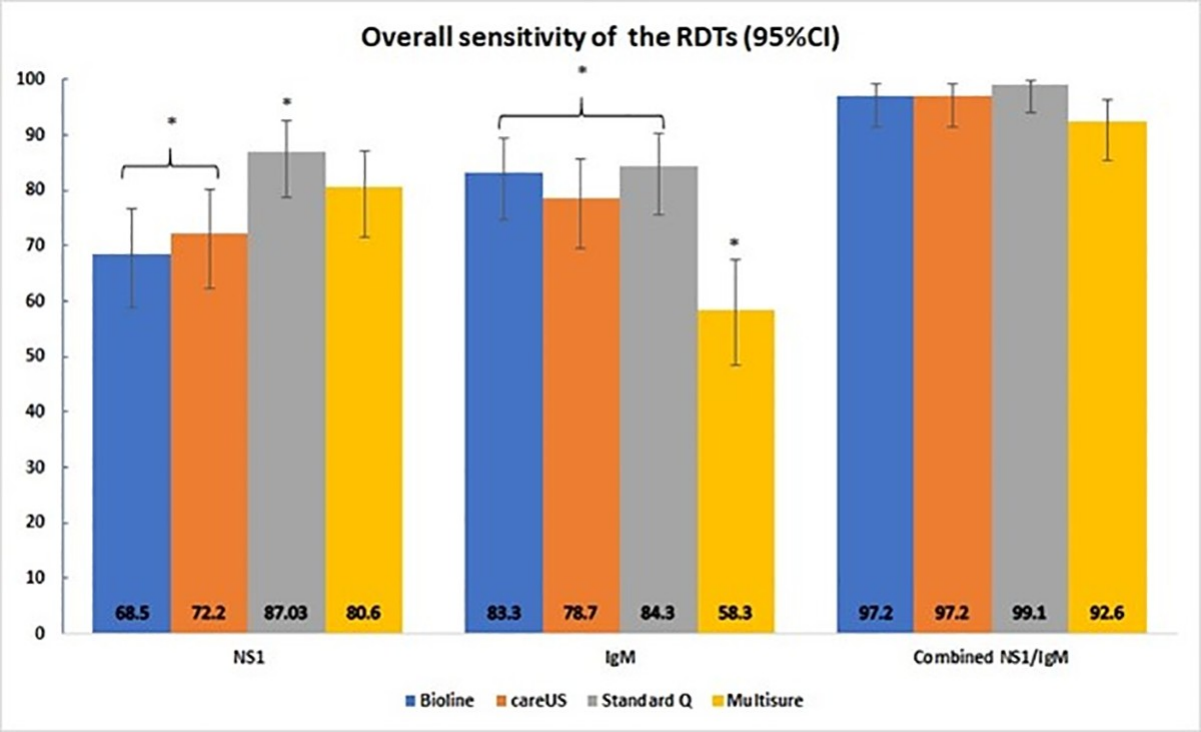
Overall sensitivity of the RDTs. The RDTs are colour coded and grouped into NS1, IgM or combined NS1/IgM categories. The bar charts indicate the sensitivity estimates and the solid black lines indicate the 95% confidence intervals for those estimates. (*) indicates a significant difference between 2 or more RDTs within each group.

Four kits were put through this phase of evaluation. Standard Q had the highest NS1 sensitivity at 87.0%, which is significantly higher than Bioline and careUS. Standard Q also had the highest IgM sensitivity at 84.3%, whereas Multisure had significantly lower IgM sensitivity compared to the other three RDTs. When we combined the results for NS1 and IgM detection, the sensitivity of the RDTs improved substantially. Standard Q had the highest sensitivity at 99.1% and was significantly higher than Multisure with a p-value of 0.04. The overall sensitivity of the RDTs are summarized in Fig 2.

All RDTs were highly specific for dengue NS1 at 100%. For dengue IgM, the specificity of Multisure was 96.7% whereas the other three RDTs were highly specific at 100%. However, this difference was not statistically significant.

The positive predictive values (PPVs) for the NS1, IgM and combined NS1/IgM parameters were all above 95%. In contrast, the negative predictive values (NPVs) ranged from 46.9% to 68.2% for NS1 and 39.2% to 63.8% for IgM. The NPVs range increased to 78.4% to 96.8% for combined NS1/IgM. Standard Q demonstrated the highest NPVs for NS1, IgM and combined NS1/IgM. The PPVs and NPVs of the RDTs are summarized in Table 5.

**Table 5:** Positive and negative predictive values of the RDTs to dengue.

Dengue panel	Test	% PPV (95% CI)	% NPV (95% CI)
**NS1**	Bioline	100 (95.1–100)	46.9 (35.2–58.9)
	careUS	100 (95.3–100)	50.0 (37.7–62.3)
	Standard Q	100 (96.1–100)	68.2 (53.4–80.0)
	Multisure	100 (95.8–100)	58.8 (45.2–71.2)
**IgM**	Bioline	100 (95.9–100)	62.5 (48.4–74.8)
	careUS	100 (95.7–100)	56.6 (43.3–69.0)
	Standard Q	100 (95.9–100)	63.8 (49.5–76.0)
	Multisure	98.4 (91.7–99.7)	39.2 (28.9–50.6)
**Combined NS1/IgM**	Bioline	100 (96.5–100)	90.9 (76.4–96.9)
	careUS	100 (96.5–100)	90.9 (76.4–96.9)
	Standard Q	100 (96.5–100)	96.8 (83.8–99.4)
	Multisure	99.0 (94.6–99.8)	78.4 (62.8–88.6)

### 3.3 Phase 2 –The effect of time on the RDTs’ sensitivity

**Fig 3:**
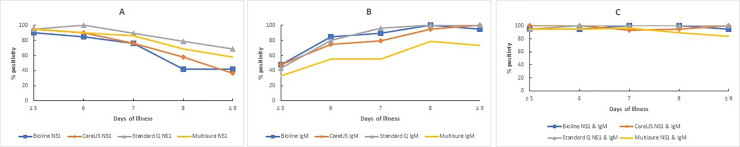
Effect of time (number of days of illness) on RDT sensitivity for (A) NS1, (B) IgM and (C) combined NS1/IgM.

High number of NS1 positives were detected in the early phase of illness and subsequently declined over time. careUS saw the steepest decline in NS1 detection from 95.2% at days of illness ≤ 5 to 36.8% at days of illness ≥ 9 whereas Standard Q declined the least from 95.2% to 68.4% (Fig 3A). IgM was gradually detected as the illness progressed from days ≤ 5 to days ≥ 9. Standard Q had the largest increment in IgM detection rate from 42.92% at days of illness ≤ 5 to 100% at days of illness ≥ 9 whereas Multisure had the least increment in IgM detection rate from 33.3% to 73.7% (Fig 3B). In combined NS1/IgM, the sensitivity of all the RDTs were at least 84.2% across the days of illness (Fig 3C). The detection rate for NS1, IgM and combined NS1/IgM at each day of illness are available as supplementary data in the annex.

## 4. Discussion

In this study, we evaluated six dengue RDTs for their diagnostic accuracy and capability in detecting recent dengue infection. The RDT with the highest diagnostic accuracy and capability can be used for dengue testing thereby enhancing Singapore’s vector control effort.

In phase 1, Truquick and Quickprofile cross reacted with both zika and chikungunya virus supernatant at relatively low titre of 10^2^ to 10^3^ pfu/ml. The low specificity will potentially result in false dengue positive in countries where zika, chikungunya and dengue virus co-circulate and hamper the efforts in disease control, patient management and etiological investigation.

The phase 2 results showed that Standard Q had the highest overall diagnostic accuracy for NS1 and IgM using specimens collected in Singapore, which have low incidences or no reports of other related flaviviruses such as Yellow fever and Japanese encephalitis.

When IgM results were analysed in combination with NS1 results, the sensitivity of all the RDTs in determining a recent dengue infection reached at least 92.6%. This observation was consistent with several studies reported [23–25]. However, a single IgM result can be indicative of past dengue or flaviviruses infection and further testing is needed for definitive dengue diagnosis [23].

Positive and negative predictive values are indicative of the RDT’s diagnostic capability in clinical setting where the true disease status of a patient is unknown. These values indicate the probability that the RDT will provide a correct diagnosis [26]. All the RDTs evaluated had similar high PPVs (98.4% to 100%), suggesting that their probability to correctly confirm a dengue infection was high when the test results were positive. However, Standard Q had the highest NPV across all 3 dengue panels. This suggest that out of the RDTs evaluated, Standard Q had the highest probability to correctly confirm a non-dengue infection when the test results were negative.

The effect of days of illness on the RDTs sensitivity to NS1, IgM and combined NS1/IgM were consistent with several studies reported [10–12,25]. Our data is in agreement with a previous study where NS1 is more likely to be detected in the early phase of illness and IgM at the later phase of illness [27]. In combined NS1/IgM, the sensitivity of all the RDTs were consistently high, suggesting their ability to detect recent dengue infection from acute to convalescent phase of the infection.

The specimen type used for this evaluation limited us. Out of the 108 pre-characterised specimens, 91.67% were DENV-2 hence, the dengue NS1 sensitivity for DENV-1, 3 and 4 may not be representative in this study. As described in a 2014 study, the Bio-Rad Platelia™ Dengue NS1 antigen capture kit was reported to have a lower sensitivity for DENV-4 circulating in Roraima, Brazil and for DENV-2 circulating in Southeast Asia [28]. This suggested that there are structural differences between NS1 of different dengue serotypes, which might be a potential issue in Singapore where all four serotypes are known to co-circulate. The specimens used to determine specificity in this study were limited. More specimens from other non-dengue febrile illnesses, related flavivirus and other arthropod borne infections such as Yellow fever, Japanese encephalitis, West Nile, Rickettsia and Malaria should be included for testing. Lastly, the visual reading of test results at times can still be subjective even for trained analysts. Future studies with additional inter-rater agreement would strengthen diagnostic accuracy.

In March 2020, Singapore reported two cases of COVID-19 positive patients who were misdiagnosed as dengue serology positive. The investigation revealed that the initial dengue serology results tested by careUS and Bioline were false positives [29]. Therefore, test results must be interpreted with symptoms, especially with IgM tests, which could cross react with other immunological responses. Failing to consider other diseases because of a positive dengue serology results could lead to serious implications for both the patient and public health.

Consistent intra- and inter- lot performance are important indicators of the RDT’s quality. The Environmental Health Institute laboratory encountered intra-lot IgM variability when using a certain brand for routine clinical testing. Therefore, intra-/inter- lot variability should be tested when carrying out such evaluation to minimize the possibility of misdiagnosis as highlighted previously.

## 5. Conclusion

In this study, we evaluated six dengue RDTs for their diagnostic accuracy and capability in detecting recent dengue infections. Truquick and Quickprofile cross reacted with zika and chikungunya virus while none of the RDTs cross reacted with zika IgM. Standard Q showed a high degree of sensitivity and specificity in the detection of dengue NS1 and IgM biomarkers and had the highest positive and negative predictive values, demonstrating its utility in enhancing dengue disease surveillance and control programmes. Further studies are required to assess the usefulness of the RDTs in other epidemiology settings. Clinicians must be aware that a negative result does not rule out dengue. Conversely, a positive dengue antibody result must be interpreted with symptoms and does not rule out the possibility of other diseases.

## Supporting information

S1 Fig Overall sensitivity of the RDTs. The RDTs are colour coded and grouped into NS1, IgM or combined NS1/IgM categories. The bar charts indicate the sensitivity estimates and the solid black lines indicate the 95% confidence intervals for those estimates. (*) indicates a significant difference between 2 or more RDTs within each group.
(DOCX)

S2 Fig Effect of time (number of days of illness) on RDT sensitivity for (A) NS1, (B) IgM and (C) combined NS1/IgM.
(DOCX)

S1 Table. Positive and negative predictive values of the RDTs to dengue (XLSX)

S1 Annex (DOCX)
