# Supplementary material for: Rapid diagnostic tests for the detection of recent dengue infections: An evaluation of six kits on clinical specimens
Source: PLoS One. 2021 Apr 1;16(4):e0249602. doi: 10.1371/journal.pone.0249602 (PMC8016316; doi:10.1371/journal.pone.0249602)
Supplement: S2 Fig — Effect of time (number of days of illness) on RDT sensitivity for (A) NS1, (B) IgM and (C) combined NS1/IgM. (DOCX) [file pone.0249602.s002.docx]

| **Test Panel** | **Sensitivity (%) at days of illness** | | | | |
| --- | --- | --- | --- | --- | --- |
|  | **≤ 5 days (n=21)** | **6 day (n=20)** | **7 day (n=29)** | **8 day (n=19)** | **≥ 9 days (n=19)** |
| Bioline NS1 | 90.5 [19/21] | 85 [17/20] | 75.9 [22/29] | 42.1 [8/19] | 42.1 [8/19] |
| CareUS NS1 | 95.2 [20/21] | 90 [18/20] | 75.9 [22/29] | 57.9 [11/19] | 36.8 [7/19] |
| Standard Q NS1 | 95.2 [20/21] | 100 [20/20] | 89.7 [26/29] | 78.9 [15/19] | 68.4 [13/19] |
| Multisure NS1 | 95.2 [20/21] | 90 [18/20] | 86.2 [25/29] | 68.4 [13/19] | 57.9 [11/19] |
|  | | | | | |
| Bioline IgM | 47.6 [10/21] | 85 [17/20] | 89.7 [26/29] | 100 [19/19] | 94.7 [18/19] |
| CareUS IgM | 47.6 [10/21] | 75 [15/20] | 79.3 [23/29] | 94.7[18/19] | 100 [19/19] |
| Standard Q IgM | 42.9 [9/21] | 80 [16/20] | 96.6 [28/29] | 100 [19/19] | 100 [19/19] |
| Multisure IgM | 33.3 [7/21] | 55 [11/20] | 55.2 [16/29] | 78.9 [15/19] | 73.7 [14/19] |
|  | | | | | |
| Bioline NS1 & IgM | 95.2 [20/21] | 95 [19/20] | 100 [29/29] | 100 [19/19] | 94.7 [18/19] |
| CareUS NS1 & IgM | 100 [21/21] | 100 [20/20] | 93.1 [27/29] | 94.7 [18/19] | 100 [19/19] |
| Standard Q NS1 & IgM | 95.2 [20/21] | 100 [20/20] | 100 [29/29] | 100 [19/19] | 100 [19/19] |
| Multisure NS1 & IgM | 95.2 [20/21] | 95 [19/20] | 96.6 [28/29] | 89.5 [18/19] | 84.2 [16/19] |
