# Supplementary material for: Rapid diagnostic tests for the detection of recent dengue infections: An evaluation of six kits on clinical specimens
Source: PLoS One. 2021 Apr 1;16(4):e0249602. doi: 10.1371/journal.pone.0249602 (PMC8016316; doi:10.1371/journal.pone.0249602)
Supplement: S1 Annex — (DOCX) [file pone.0249602.s004.docx]

**Annex**

**Supplementary Data**

| **Dengue panels** | **Test** | **% Sensitivity (95% CI)** | **% Specificity (95% CI)** | **% PPV**  **(95% CI)** | **% NPV (95% CI)** |
| --- | --- | --- | --- | --- | --- |
| **NS1**  NS1: 108  Non-NS1: 30 | Bioline | 68.5* [74/108]  (58.8 – 76.9) | 100 [30/30]  (85.9 – 100) | 100  (95.1 – 100) | 46.9  (35.2 – 58.9) |
|  | careUS | 72.2* [78/108]  (62.3 – 80.2) | 100 [30/30]  (85.9 – 100) | 100  (95.3 – 100) | 50.0  (37.7 – 62.3) |
|  | Standard Q | 87.03* [94/108]  (78.9 – 92.5) | 100 [30/30]  (85.9 – 100) | 100  (96.1 – 100) | 68.2  (53.4 – 80.0) |
|  | Multisure | 80.6 [87/108]  (71.6 – 87.2) | 100 [30/30]  (85.9 – 100) | 100  (95.8 – 100) | 58.8  (45.2 – 71.2) |
| (*) indicates a significant difference between 2 or more RDTs within each group. | | | | | |
| **IgM**  IgM: 108  Non-IgM: 30 | Bioline | 83.3 [90/108]  (74.7 – 89.6) | 100 [30/30]  (85.9 – 100) | 100  (95.9 – 100) | 62.5  (48.4 – 74.8) |
|  | careUS | 78.7 [85/108]  (69.6 – 85.8) | 100 [30/30]  (85.9 – 100) | 100  (95.7 – 100) | 56.6  (43.3 – 69.0) |
|  | Standard Q | 84.3 [91/108]  (75.7 – 90.3) | 100 [30/30]  (85.9 – 100) | 100  (95.9 – 100) | 63.8  (49.5 – 76.0) |
|  | Multisure | 58.3* [63/108]  (48.4 – 67.6) | 96.7 [29/30]  (80.9 -99.8) | 98.4  (91.7 – 99.7) | 39.2  (28.9 – 50.6) |
| (*) indicates a significant difference between 2 or more RDTs within each group. | | | | | |
| **IgG**  IgG: 79  Non-IgG: 30 | Bioline | 64.6 [51/79]  (52.9 – 74.8) | 100 [30/30]  (85.9 – 100) | 100  (93.0 – 100) | 51.7  (39.2 – 64.1) |
|  | careUS | 68.4 [54/79]  (56.8 – 78.1) | 100 [30/30]  (85.9 – 100) | 100  (93.4 – 100) | 54.5  (41.5 – 67.0) |
|  | Standard Q | 58.2 [46/79]  (46.6 – 69.1) | 100 [30/30]  (85.9 – 100) | 100  (92.3 – 100) | 47.6  (35.8 – 59.7) |
|  | Multisure | 11.4* [9/79]  (5.6 – 21.0) | 100 [30/30]  (85.9 – 100) | 100  (70.1 – 100) | 30.0  (21.9 – 39.6) |
| (*) indicates a significant difference between 2 or more RDTs within each group. | | | | | |
| **Combined NS1/IgM**  Dengue: 108  Non-dengue: 30 | Bioline | 97.2 [105/108]  (91.5 – 99.3) | NA | 100  (96.5 – 100) | 90.9  (76.4 – 96.9) |
|  | careUS | 97.2 [105/108]  (91.5 – 99.3) | NA | 100  (96.5 – 100) | 90.9  (76.4 – 96.9) |
|  | Standard Q | 99.1* [107/108]  (94.2 – 99.95) | NA | 100  (96.5 – 100) | 96.8  (83.8 – 99.4) |
|  | Multisure | 92.6*[100/108]  (85.5 – 96.5) | NA | 99.0  (94.6 – 99.8) | 78.4  (62.8 – 88.6) |
| (*) indicates a significant difference between 2 or more RDTs within each group. | | | | | |

**Table 6. Diagnostics accuracy of the RDTs**

| **Test Panel** | **Sensitivity (%) at days of illness** | | | | |
| --- | --- | --- | --- | --- | --- |
|  | **≤ 5 days (n=21)** | **6 day (n=20)** | **7 day (n=29)** | **8 day (n=19)** | **≥ 9 days (n=19)** |
| Bioline NS1 | 90.5 [19/21] | 85 [17/20] | 75.9 [22/29] | 42.1 [8/19] | 42.1 [8/19] |
| CareUS NS1 | 95.2 [20/21] | 90 [18/20] | 75.9 [22/29] | 57.9 [11/19] | 36.8 [7/19] |
| Standard Q NS1 | 95.2 [20/21] | 100 [20/20] | 89.7 [26/29] | 78.9 [15/19] | 68.4 [13/19] |
| Multisure NS1 | 95.2 [20/21] | 90 [18/20] | 86.2 [25/29] | 68.4 [13/19] | 57.9 [11/19] |
|  | | | | | |
| Bioline IgM | 47.6 [10/21] | 85 [17/20] | 89.7 [26/29] | 100 [19/19] | 94.7 [18/19] |
| CareUS IgM | 47.6 [10/21] | 75 [15/20] | 79.3 [23/29] | 94.7[18/19] | 100 [19/19] |
| Standard Q IgM | 42.9 [9/21] | 80 [16/20] | 96.6 [28/29] | 100 [19/19] | 100 [19/19] |
| Multisure IgM | 33.3 [7/21] | 55 [11/20] | 55.2 [16/29] | 78.9 [15/19] | 73.7 [14/19] |
|  | | | | | |
| Bioline NS1 & IgM | 95.2 [20/21] | 95 [19/20] | 100 [29/29] | 100 [19/19] | 94.7 [18/19] |
| CareUS NS1 & IgM | 100 [21/21] | 100 [20/20] | 93.1 [27/29] | 94.7 [18/19] | 100 [19/19] |
| Standard Q NS1 & IgM | 95.2 [20/21] | 100 [20/20] | 100 [29/29] | 100 [19/19] | 100 [19/19] |
| Multisure NS1 & IgM | 95.2 [20/21] | 95 [19/20] | 96.6 [28/29] | 89.5 [18/19] | 84.2 [16/19] |

**Table 7. Effect of time (number of days of illness) on RDT sensitivity for NS1, IgM and combined NS1/IgM.**
